# Adjacent segment disease induced by spinal tophus: a case report

**DOI:** 10.3389/fsurg.2025.1545557

**Published:** 2025-05-14

**Authors:** Chenglong Wang, Hongjun Liu, Shuangquan Gong, Yu Ye, Liqiang Cui, Dengshang Liu, Sen Li, Shiming Xie

**Affiliations:** ^1^ Spinal Surgery Department, Mianyang Orthopaedic Hospital, Mianyang, Sichuan, China; ^2^ Division of Spine Surgery, Department of Orthopedic Surgery, Nanjing Drum Tower Hospital, Affiliated Hospital of Medical School, Nanjing University, Nanjing, Jiangsu, China

**Keywords:** tophus, adjacent segment disease, monosodium urate crystal deposition, uric acid, case report

## Abstract

Tophus, a hallmark feature of chronic gout, typically develops in the joints of the extremities, skin, and mucosal tissues. Although several case reports have described spinal tophus deposition leading to spinal stenosis and radiculopathy, documented cases of lumbar adjacent segment disease (ASD) caused by monosodium urate crystal (MUC) deposition remain exceedingly rare. Here, we report the case of a 56-year-old male who underwent L4-S1 lumbar fusion surgery two years prior for L4-S1 disc herniation with radiculopathy. The patient presented with recurrent low back pain, radiculopathy, and systemic tophus involvement. Magnetic resonance imaging (MRI) T2-weighted sequences revealed a hypointense signal at the posterior margin of the L3 vertebral body, while computed tomography (CT) demonstrated a hyperdense lesion. These radiographic findings suggested dural sac compression and spinal stenosis, though the definitive etiology warranted further evaluation. Surgical intervention involved excision of the L3 posterior lesion and extension of the fusion construct from L4-S1 to L2-S1 based on the existing L4-S1 instrumentation for spinal stabilization. Histopathological examination confirmed extensive MUC deposition. This report details the clinical presentation, imaging characteristics, pathological findings, surgical management, and potential pathogenic mechanisms underlying this rare complication.

## Introduction

Gout is a metabolic disorder of purine metabolism, clinically characterized by recurrent acute and chronic arthritis, with tophus formation marking the chronic phase (1, 2). Tophi typically develop in areas of poor blood circulation and lower temperatures, such as the ear helix and first metatarsophalangeal joint, often leading to articular cartilage/bone erosion and periarticular soft-tissue fibrosis (3, 4). Although rare case reports describe lumbar radiculopathy secondary to spinal tophus (5–8), no published cases to our knowledge document adjacent segment disease (ASD) caused by tophus formation following lumbar fusion surgery. Here, we report a unique case of post-fusion ASD involving tophus-induced dural compression and spinal stenosis at the adjacent level. The lesion was surgically excised via a posterior approach, with extension of the fusion construct to restore spinal stability. Histopathological examination confirmed extensive monosodium urate crystal (MUC) deposition within degenerated nucleus pulposus tissue.

## Case report

**Figure 1:**
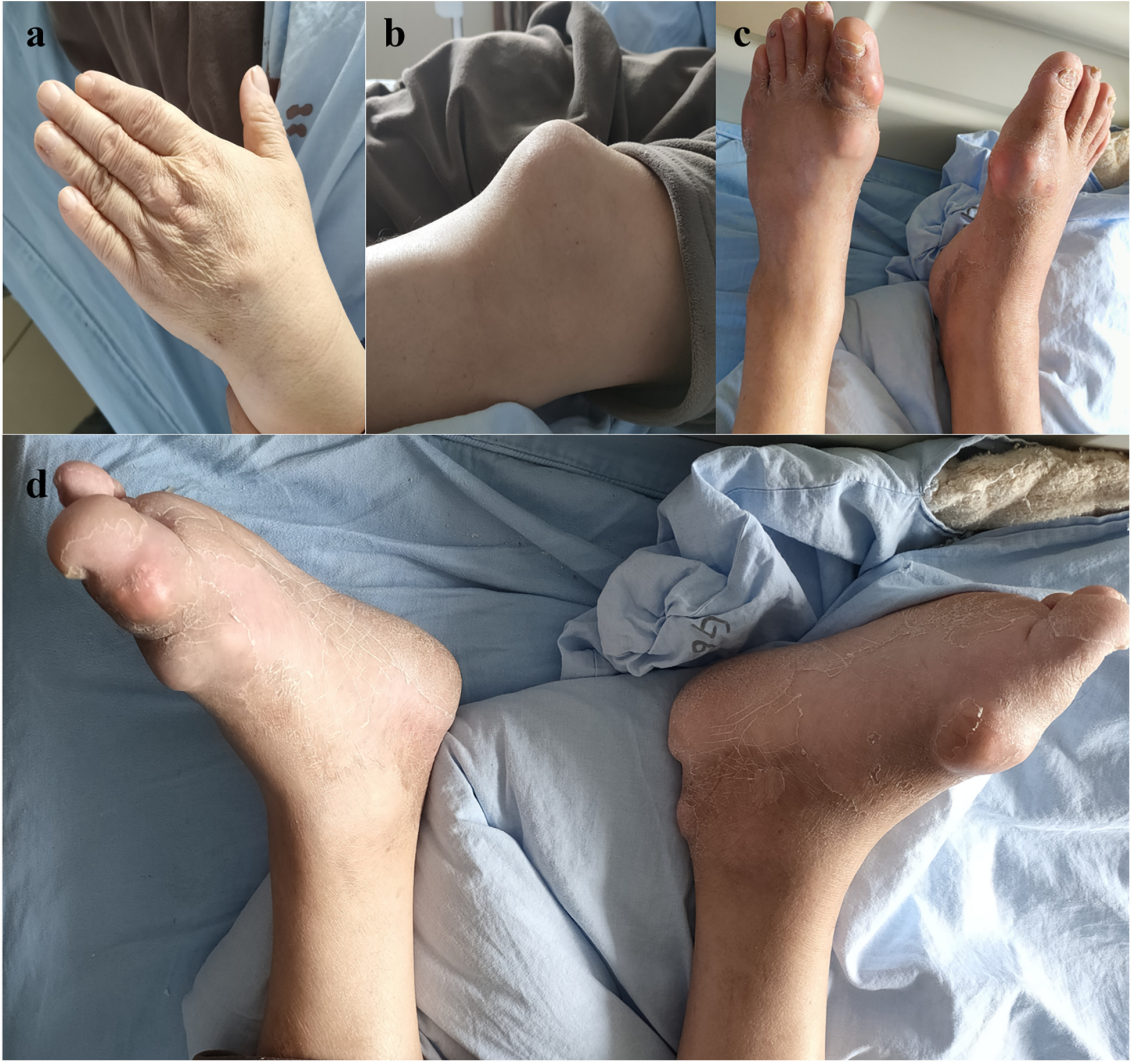
Multiple tophi were identified throughout the body: **(a)** left hand; **(b)** left knee; **(c,d)** both feet.

**Figure 2:**
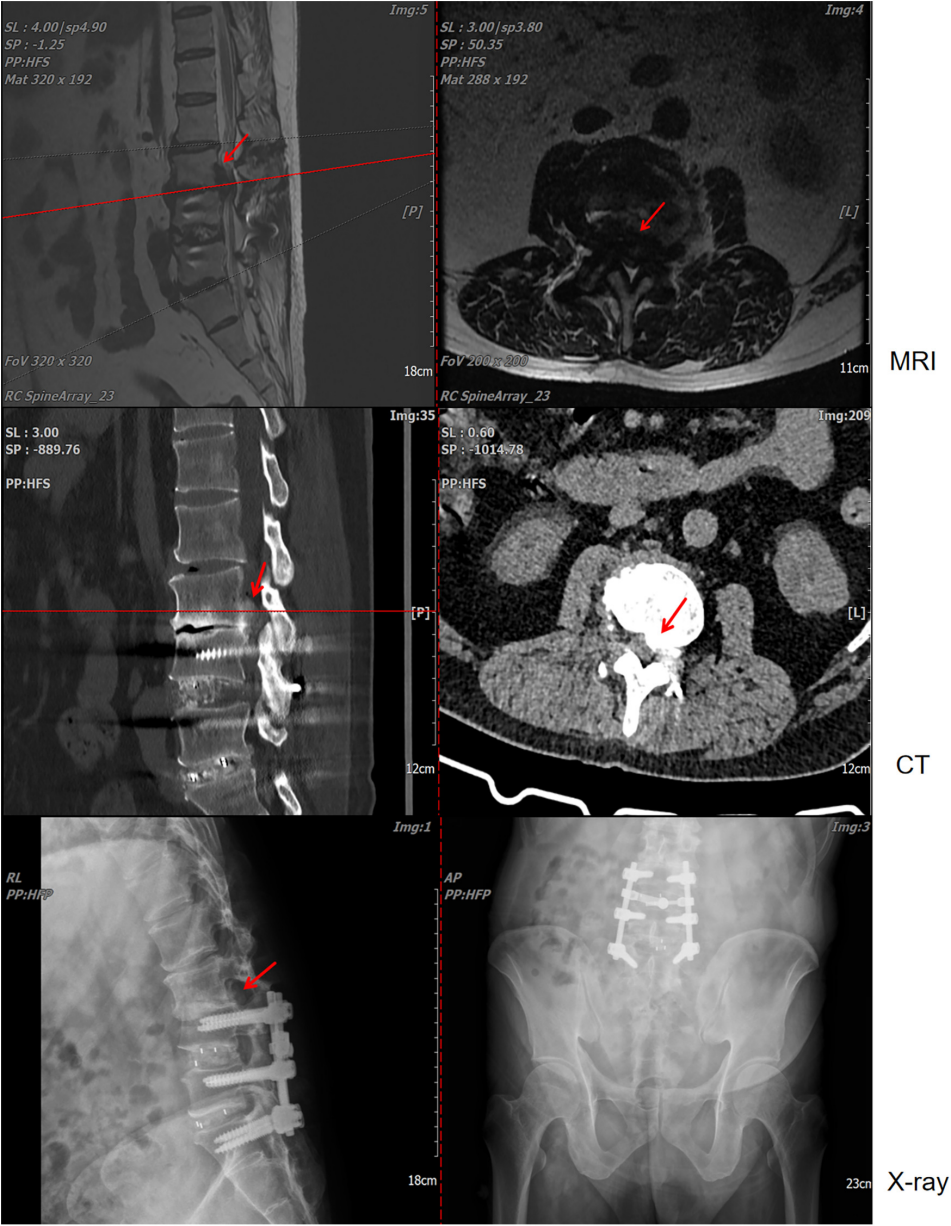
Lumbar magnetic resonance imaging (MRI), computed tomography (CT), and x-ray examinations revealed L4-S1 lumbar fusion with screw rod internal fixation. Concurrently, imaging indicated that the lesion at the posterior edge of the L3 vertebral body exerted compression on the dural sac, resulting in secondary spinal stenosis.

A 56-year-old male patient with a history of L4-S1 discectomy followed by instrumented fusion at an external institution two years prior presented with a four-month history of recurrent low back pain and left lower extremity radiculopathy. Over the past 20 days, the symptoms had progressed significantly, manifesting as notable neurogenic intermittent claudication. Physical examination revealed grade 4 strength in the left rectus femoris muscle with diminished deep tendon reflexes in both the right patellar and Achilles tendons. The patient had a history of gout for over 20 years, with clinical manifestations including diffuse swelling (Figure 1) and pain in the acute-phase in multiple joints of the extremities. Previously, the patient received an irregular/intermittent treatment regimen at another hospital, which included: prednisone (0.5 mg/kg/day, orally), celecoxib (0.2 g, twice daily, orally), colchicine (an initial dose of 1 mg, orally, followed by 0.5 mg after 1 h, and then 0.5 mg every 2–3 h until symptom relief), and febuxostat (40 mg, once daily, orally). The Visual Analogue Scale (VAS), Japanese Orthopaedic Association (JOA) and Oswestry Disability Index (ODI) scores for back pain were recorded as 7, 14 and 27 (60%), respectively. Serum analysis revealed uric acid (UA) and creatinine levels of 601 μmol/L and 120 μmol/L, respectively. Hematological parameters showed a white blood cell (WBC) count of 6.64 × 10^9^/L, with an erythrocyte sedimentation rate (ESR) of 10 mm/h and C-reactive protein (CRP) level of 9.4 mg/L (Table 1). Radiological examination demonstrated hypodense signal intensity at the posterior margin of the L3 vertebral body on T2-weighted MRI sequences, with corresponding hyperdense signal intensity on CT imaging, resulting in dural sac compression and consequent spinal stenosis (Figure 2). Bone mineral density assessment yielded a *T*-score of −2.1, consistent with osteoporosis.

**Table 1:** Presents the results of laboratory tests at different time nodes, mainly focusing on uric acid, creatinine, white blood cells (WBC), erythrocyte sedimentation rate (ESR), and C-reactive protein (CRP).

Time nodes	Uric acid (μmol/L)	Creatinine (μmol/L)	WBC ×10^9^/L	CRP (mg/L)	ESR (mm/h)
1 month before surgery	551	75	6.79	6.8	11
2 days before surgery	601	120	6.64	9.4	10
1 day after surgery	588	114	13.4	8.3	14
7 days after surgery	448	94	9.69	89	70

**Figure 3:**
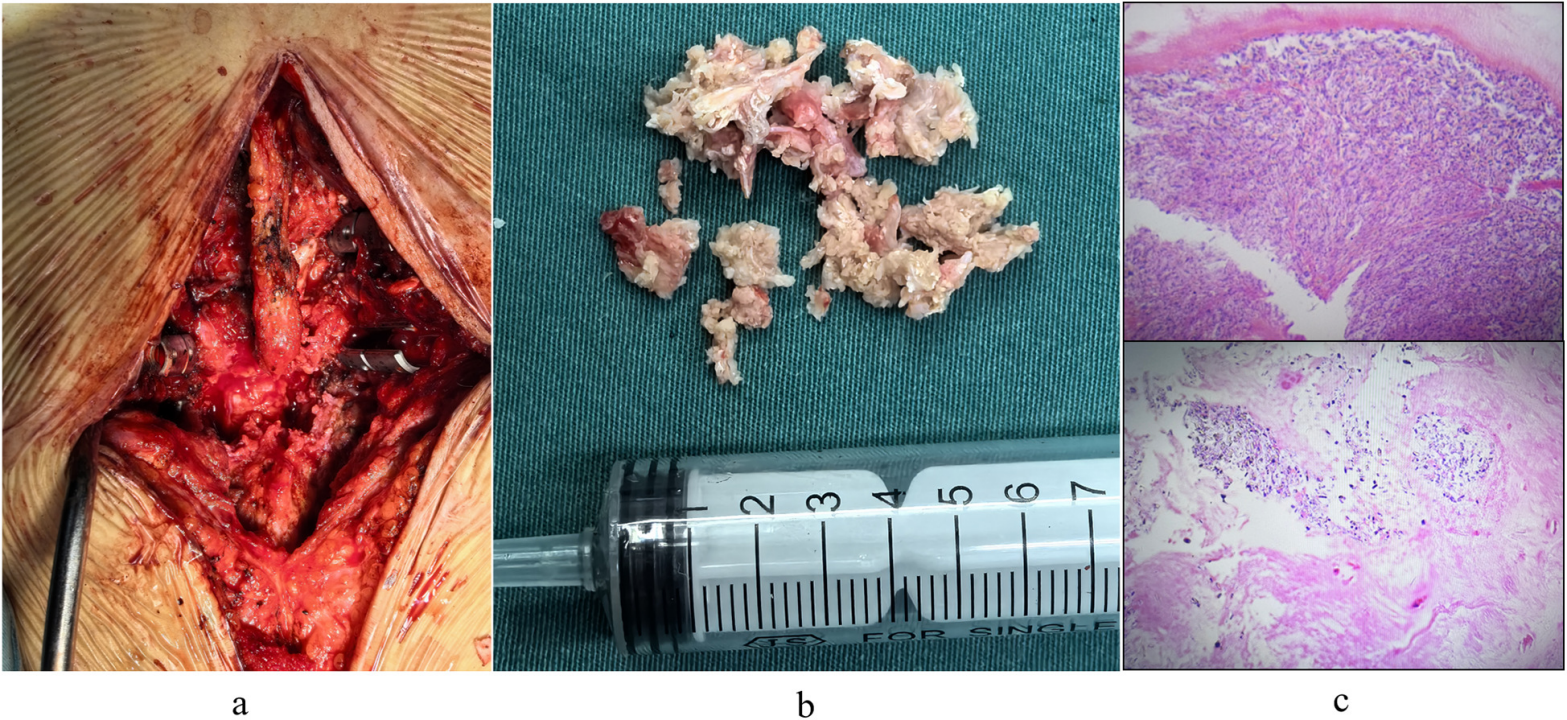
**(a)** surgical excision of chalk-like lesions. **(b)** Intraoperative images captured post-lesion removal and decompression. **(c)** Pathological staining revealed that the degenerated nucleus pulposus tissue was intermixed with a substantial quantity of monosodium urate crystal (MUC) deposits.

The patient underwent lesion excision at the posterior margin of the L3 vertebral body, spinal decompression, and pedicle screw fixation under general anesthesia. A revision operation was performed to extend fusion and internal fixation from L4-S1 to L2-S1 (Figure 3a). Due to concomitant osteoporosis-likely attributable to suboptimal oral hormone therapy during prior acute gout attacks-vertebral reinforcement with cement was performed. Intraoperative examination revealed that the L3 lesion consisted of herniated nucleus pulposus tissue intermingled with a substantial calcified, chalky-white mass (Figure 3b), which was completely excised. Histopathological analysis of the chalk-like specimens via hematoxylin and eosin (HE) staining confirmed extensive MUC deposits (Figure 3c). Postoperatively, the patient received systematic gout management and rehabilitation. Given the patient's history of disorganized and suboptimal gout treatment, along with elevated serum uric acid and creatinine levels (non-acute phase), we initiated a combined urate-lowering therapy with febuxostat (40 mg once daily) and benzbromarone (50 mg once daily). By postoperative day 7, the serum uric acid level had decreased to 448 μmol/L, and creatinine levels to 94 μmol/L (Table 1).

**Figure 4:**
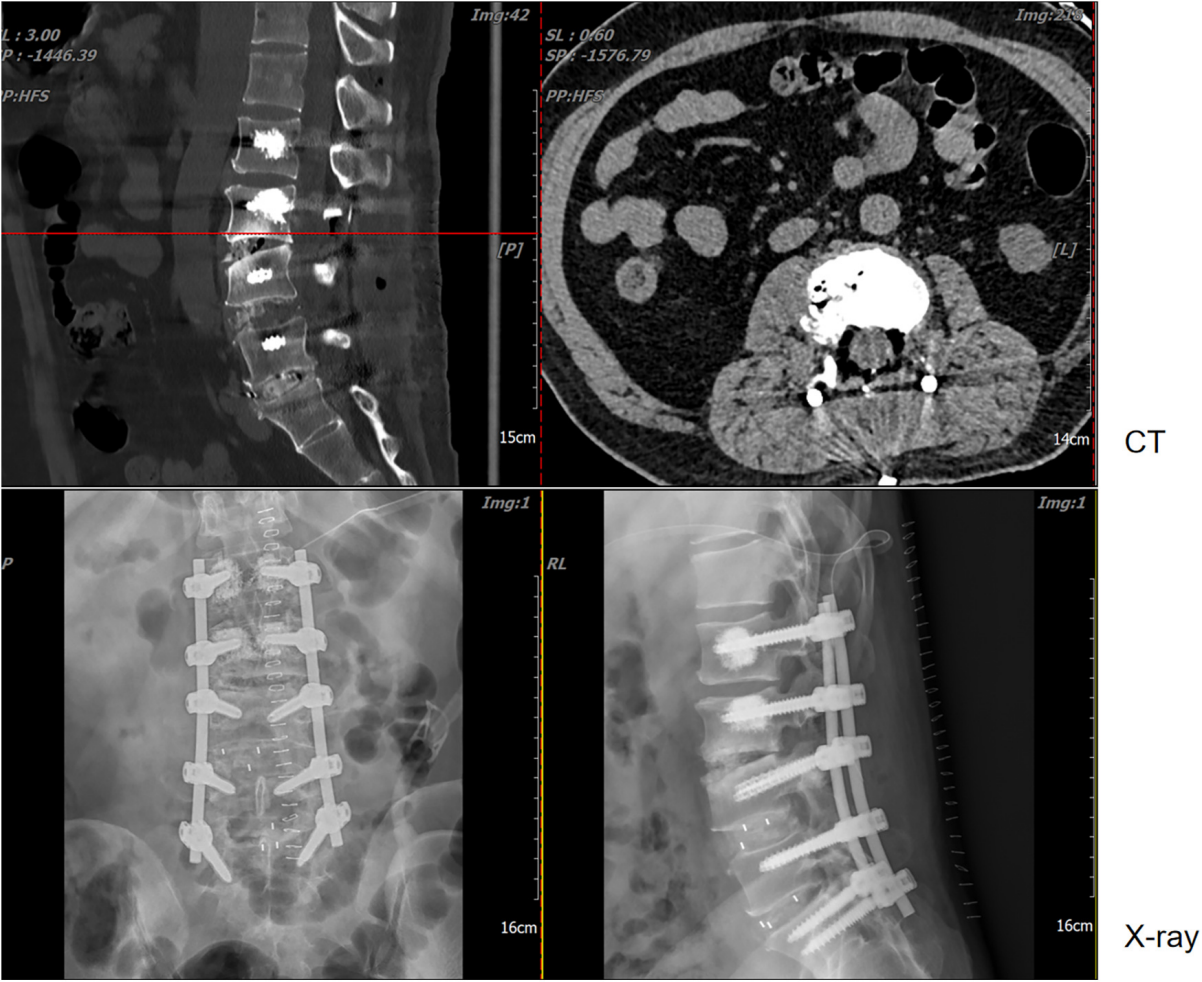
Postoperative CT and anteroposterior and lateral x-ray images.

The patient was satisfied with the treatment process, mainly because the procedure resolved the uncomfortable symptoms that patient had suffered. At 2 weeks post-surgery, the VAS, JOA, and ODI scores were recorded as 3, 25, and 10 (20%), respectively. Follow-up lumbar x-ray and CT examinations revealed no evidence of spinal tophus recurrence or screw loosening (Figure 4).

## Discussion

Elevated serum uric acid levels are well established to cause monosodium urate crystal deposition in periarticular tissues, including synovium, subchondral bone, and articular cartilage. Although spinal tophus-induced lumbar radiculopathy has been documented in rare cases, nerve root compression in adjacent segments secondary to tophus formation following spinal fusion represents an exceptionally uncommon clinical presentation.

We sought to investigate the causative mechanisms underlying this condition. The etiology and pathogenesis of MUC deposition in the spinal skeleton remain poorly understood, having received limited research attention. However, limited evidence suggests that spinal degenerative changes, tissue necrosis, or prior trauma may contribute to this rare phenomenon (5, 6, 9). The predilection for peripheral joint involvement in gout is primarily attributed to both the reduced solubility of MUC at lower temperatures and the propensity for tophus formation in avascular tissues (10). These joints demonstrated an extensive range of motion. In this case, the previous fusion surgery at the L4-S1 level constrained lumbar spine mobility, thereby increasing mechanical load on the adjacent segment (the L3-4 disc). This compensatory mechanism is commonly associated with a higher incidence of adjacent segment pathology. And then, reduced blood pH diminished plasma protein binding capacity, while concurrent trauma exacerbated MUC precipitation-both factors promoting accelerated tophus formation. The patient's pre-existing gout history and prior lumbar fusion surgery collectively established the pathophysiological conditions necessary for spinal tophus development in this clinical scenario (8, 10–12). Furthermore, in the present case, degenerative changes in the spine were observed, which may have facilitated spinal tophus formation and contributed to vertebral body and intervertebral disc involvement. These findings are consistent with prior studies demonstrating that crystal deposits frequently occur in multiple spinal structures, including vertebral bodies, pedicles, laminae, ligamentum flavum, interosseous cartilage, intervertebral discs, and the epidural space (5, 12, 13). Notably, existing evidence suggests that all spinal segments in gout patients are susceptible to involvement, with the lumbar spine (particularly the L4-5 level) being the most frequently affected site (7). In this case, however, the L3-4 level was implicated. Collectively, we propose that gout onset at the L3-4 spine in this patient likely resulted from a combination of factors, including a history of gout, lumbar degeneration, prior lumbar trauma, and compensatory increases in pressure and motion at adjacent segments following lumbar fusion.

Spinal gout represents a diagnostic challenge due to its presentation as an epidural space-occupying lesion with lower back symptoms, requiring differentiation from other pathologies, including disc herniation, spinal infections, and neoplastic lesions. Notably, in patients with prior lumbar surgery, adjacent segment disease is often attributed to nucleus pulposus protrusion. Nevertheless, clinicians must thoroughly evaluate the patient's medical history, correlate imaging findings, and interpret histopathological results to ensure accurate diagnosis and guide optimal therapeutic intervention.

## Data Availability

The original contributions presented in the study are included in the article/Supplementary Material, further inquiries can be directed to the corresponding authors.
